# The Greatest Doctor

**Published:** 1871-04

**Authors:** 


### THE GREATEST DOCTOR

Is Nature ; just let her have her way, and she will cure three fourths of all ordinary sickness without the cost of a dollar. In one important respect the human machine, built by the power, and wisdom, and benevolence of the INFINITE ONE, passes any structure of human hands by a distance immeasurable, — it mends itself, it repairs the injuries received from outsiders with wonderful promptitude and perfectness. Cut a gash in the arm ; let it alone, and it will get well. Let a man make a fool of himself by getting drunk or eating twice as much as he ought to have done, and although Nature has been outraged, the body shattered, the mind befuddled, and the man degraded, just give Nature a chance at him with a ten hour's sleep, and she will make it

"ALL RIGHT IN THE MORNING."

But the trouble is, man is such a fool, instead of letting "the great doctor" have his way, he takes to "arguing the case," when he gets sick, and thus reasons : "When I was well, I could eat heartily ; now if I can only eat heartily, I shall be well ; " and right away he orders a splendid dinner, and to give him an appetite, he drinks brandy first, peppers his vegetables, plasters his meats with mustard, fills his plate with Worcester sauce, and "tops off" with absinthe, champagne, or "rotgut whiskey." No wonder people die at forty-five instead of a hundred years.

The fact should be borne in mind by every reader of cultivation and intelligence, that three fourths of the ordinary sickness of men would be safely, promptly, and efficiently cured by rest in a warm bed, in a cool room, with a clean skin, and eating only when really hungry; eating in the day-time, at six hour's intervals, of two or three kinds of the plainest food, in small quantities, for a day or two.

